# Nomogram for predicting prolonged postoperative ileus after laparoscopic low anterior resection for rectal cancer

**DOI:** 10.1186/s12957-023-03265-6

**Published:** 2023-12-11

**Authors:** Fangliang Guo, Zhiwei Sun, Zongheng Wang, Jianfeng Gao, Jiahao Pan, Qianshi Zhang, Shuangyi Ren

**Affiliations:** 1 Department of Gastrointestinal Surgery, The Second Affiliated Hospital of Dalian Medical University, Dalian, Liaoning 116023 People’s Republic of China; 2 Department of General Surgery, Shanghai Changzheng Hospital, Shanghai, 200003 People’s Republic of China

**Keywords:** Rectal cancer, Low anterior resection, Nomogram, Prolonged postoperative ileus

## Abstract

**Background:**

Prolonged postoperative ileus (PPOI) is a common complication after colorectal surgery that increases patient discomfort, hospital stay, and financial burden. However, predictive tools to assess the risk of PPOI in patients undergoing laparoscopic low anterior resection have not been developed. Thus, the purpose of this study was to develop a nomogram to predict PPOI after laparoscopic low anterior resection for rectal cancer.

**Methods:**

A total of 548 consecutive patients who underwent laparoscopic low anterior resection for mid­low rectal cancer at a single tertiary medical center were retrospectively enrolled between January 2019 and January 2023. Univariate and multivariate logistic regression analysis was performed to analyze potential predictors of PPOI. The nomogram was constructed using the filtered variables and internally verified by bootstrap resampling. Model performance was evaluated by receiver operating characteristic curve and calibration curve, and the clinical usefulness was evaluated by the decision curve.

**Results:**

Among 548 consecutive patients, 72 patients (13.1%) presented with PPOI. Multivariate logistic analysis showed that advantage age, hypoalbuminemia, high surgical difficulty, and postoperative use of opioid analgesic were independent prognostic factors for PPOI. These variables were used to construct the nomogram model to predict PPOI. Internal validation, conducted through bootstrap resampling, confirmed the great discrimination of the nomogram with an area under the curve of 0.738 (95%CI 0.736–0.741).

**Conclusions:**

We created a novel nomogram for predicting PPOI after laparoscopic low anterior resection. This nomogram can assist surgeons in identifying patients at a heightened risk of PPOI.

## Introduction

The recovery of bowel function is an important indicator following colorectal surgery, and typically, it returns to normal within 2–4 days [1, 2]. However, when the recovery of bowel function takes longer than expected, it is called prolonged postoperative ileus (PPOI). PPOI often leads to discomfort, heightened psychological distress, prolonged hospitalization, and increased hospitalization costs [3, 4].

The pathogenesis of PPOI is multifactorial and intricate [1]. Presently, risk factors such as advanced age, the use of opioid analgesics, open surgery, gastrointestinal stretch, and inflammation are considered to be key mechanisms of PPOI [5, 6]. Of note, few studies have specifically assessed the risk factors of PPOI following rectal surgery [7]. Frequent neoadjuvant irradiation, manipulation in a narrow pelvis, and creation of a diverting ileostomy may lead to a specific risk of PPOI after rectal surgery [7, 8]. The construction of PPOI prediction models can help to assess the risk of PPOI. Consequently, these models make a significant contribution to the development of strategies to reduce or prevent the occurrence of PPOI. However, to our knowledge, few studies have used nomogram prediction models to assess the risk of PPOI after colorectal surgery, especially in patients undergoing rectal surgery [5, 9, 10].

Hence, this study aimed to analyze the predictive factors for PPOI after laparoscopic anterior resection for rectal cancer and to develop a nomogram for predictive purposes.

## Patients and methods

### Study population

The data of consecutive patients with mid­low rectal cancer who underwent elective low anterior resection, between January 2019 and January 2023, were retrospectively retrieved from our prospectively collected database. To avoid confounding bias, we excluded patients with open surgery, combined multiple organ resection, and secondary surgery for postoperative recurrence.

### Operation and perioperative management

All operations were performed by an experienced surgical team, following the total mesorectal excision operation protocol. The surgical techniques were performed as described in previous reports [11, 12].

Patients were treated by the same perioperative enhanced recovery care program. Preoperative evaluation included clinical examination, serological assessment, colonoscopy, thoracoabdominal and pelvic computed tomography (CT), and pelvic magnetic resonance imaging. All patients underwent preoperative mechanical bowel preparation, and prophylactic antibiotics were administered.

Postoperative care included a clear liquid diet on postoperative day 1 and diet advancement as tolerated. In addition, the urinary catheter was removed on postoperative day 1 if there was no difficulty in urination. The utilization of postoperative use of opioid analgesics was evaluated based on the patient's postoperative visual analog scale.

### Variable and outcome definition

We adopted the definition of PPOI as proposed by Vather et al. [13]. PPOI was diagnosed when patients met at least two of the following five criteria on or after postoperative day 4: (a) nausea or vomiting, (b) inability to tolerate a solid oral diet over the last 24 h, (c) abdominal distension, (d) absence of flatus over the last 24 h, and (e) ileus noted on computed tomography (CT) scans.

The distance between the intertuberous and interspinous was measured by CT. In addition, Slice-O-matic software (version 4.3, Tomovision, Montreal, QC, Canada) was employed for body composition analysis [14]. Visceral adipose tissue area (VAT) and skeletal muscle area (SMA) were measured at the third lumbar vertebra (L3) level on the CT image, and mesorectal fat area (MFA) was measured at the level of the tip of the ischial spine. In this study, the upper quartile distributions for males and females were used as the cutoff points for VAT and MFA, respectively. Additionally, the lower quartile distributions were used as the cutoff points for SMA. Sarcopenia was defined as low-SMA and visceral obesity was defined as high VAT.

When a patient met three or more of the following five criteria, the case was classified as high surgery difficulty: narrow pelvis (intertuberous distance < 100 mm and interspinous distance < 120 mm), large MFA (> 24.14 cm^2^), low-grade tumor (tumor height ≤ 5 cm), large tumor (diameter > 5 cm), and T4 stage. Postoperative complications were classified according to the Clavien-Dindo classification [15].

### Construction and validation of the nomogram

Univariate logistic regression analysis was performed to initially assess associations of various indexes with PPOI. All indexes with a *p* value < 0.1 were included in the multivariate analysis. The variance inflation factor was calculated to ensure no collinearity among the covariates.

A nomogram based on the multivariable logistic regression was constructed. The model was validated internally using 500 bootstrap resampling to reduce overfit bias. The discrimination performance of the nomogram was assessed using receiver operating characteristic (ROC) curve analysis. Calibration curve analysis was used to assess the accuracy of the model. Additionally, decision curve analysis (DCA) was used to evaluate the clinical usefulness of the model.

### Statistical analysis

The data were analyzed by SPSS (version 25.0, IBM Corp, Armonk, New York, USA) and R software (version 4.2.1, http://www.r-project.org/). Normally distributed continuous data were analyzed by Student’s *t* test and expressed as mean (± standard deviation [SD]); non-normally distributed data were analyzed by Wilcoxon rank-sum test and expressed as median (interquartile range [IQR]). Categorical data were compared using chi-squared test or Fisher’s exact test and expressed as *n* (%). All of the statistical analyses were two-sided, and the statistical significance was set at *p* value < 0.05.

## Results

### Patient characteristics and outcomes

A total of 548 patients were included in this study, of whom 72 developed PPOI (13.1%). When comparing baseline characteristics between the two groups (Table 1), it was observed that patients in the PPOI group were older than those in the non-PPOI group (66.5 years [61–71.15] vs. 64.5 years [58–70], *p* = 0.043). In addition, more patients in the PPOI group had a history of abdominal surgery (*p* = 0.049) and hypoproteinemia (*p* = 0.011). Intergroup differences in gender, smoking history, drinking history, comorbidities, and ASA classification did not differ significantly.

**Table 1:** Clinical and anatomical characteristics of patients in the PPOI group and non-PPOI group

Variables	Non-PPOI group (*n* = 476)	PPOI group (*n* = 72)	*p*
Sex [*n* (%)]			0.261
Male	291 (61.1%)	39 (54.2%)	
Female	185 (38.9%)	33 (45.8%)	
Age [median (IQR), years]	64.5 [58–70]	66.5 [61–71.15]	0.043
Smoking history [*n* (%)]	121 (25.4%)	20 (27.8%)	0.67
Drinking history [*n* (%)]	59 (12.4%)	11 (15.3%)	0.495
Hypertension [*n* (%)]	133 (27.9%)	21 (29.2%)	0.829
Diabetes [*n* (%)]	59 (12.4%)	5 (6.9%)	0.186
Respiratory disease [*n* (%)]	48 (10.1%)	6 (8.3%)	0.642
Cardiac disease [*n* (%)]	29 (6.1%)	4 (5.6%)	1^a^
Previous abdominal surgery [*n* (%)]	80 (16.8%)	19 (26.4%)	0.049
Preoperative chemotherapy [*n* (%)]	44 (9.2%)	8 (11.1%)	0.614
Preoperative radiotherapy [*n* (%)]	37 (7.1%)	7 (9.7%)	0.438
ASA score [*n* (%)]			0.874
I	271 (56.9%)	41 (56.9%)	
II	171 (35.9%)	27 (37.5%)	
III	34 (7.1%)	4 (5.6%)	
Hypoproteinemia [*n* (%)]	36 (7.6%)	12 (16.7%)	0.011
Anemia [*n* (%)]	81 (17.0%)	19 (26.4%)	0.055
Body mass index [mean (SD), kg/m^2^]	24.35 ± 3.52	24.83 ± 3.69	0.287
Hypokalaemia [*n* (%)]	21 (4.4%)	7 (9.7%)	0.078^a^
VFA [median (IQR), cm^2^]	126.2 (80.4–182.6)	139.3 (94.8–189.1)	0.183
SMA [mean (IQR), cm^2^]	116.4 (98.0–139.0)	124.3 (103.7–148.6)	0.092
MFA [mean (IQR), cm^2^]	17.2 (11.6–23.7)	19.4 (13.1–25.7)	0.066
Tumor height [median (IQR), cm]	6 (5–8)	5.5 (5–7.9)	0.342
Interspinous distance [median (IQR), cm]	100.3 (92.7–110.2)	101.8 (94.7–116.1)	0.201
Intertuberous distance [median (IQR), cm]	116.3 (106.9–127.5)	115.5 (106.2–130.0)	0.712
Narrow pelvis [*n* (%)]	43 (9.0%)	10 (13.9%)	0.194

*ASA* American Society of Anesthesiologists Classification, *VFA* visceral adipose tissue area, *SMA* skeletal muscle area, *MFA* rectal mesenteric fat

^a^Using Fisher’s exact test

Furthermore, the operation time was longer in the PPOI group (170 min [140–201.5] vs. 155 min [126.3–182.0], *p* = 0.026). There were no statistically significant differences in terms of pathological outcomes, including tumor diameter, harvested lymph nodes, tumor differentiation, and tumor stage (Table 2).

**Table 2:** Intraoperative and pathological characteristics of patients in the PPOI group and non-PPOI group

	Non-PPOI group (*n* = 476)	PPOI group (*n* = 72)	*p*
Surgery approach [*n* (%)]			0.632
Robotic-assisted	270 (56.7%)	43 (59.7%)	
Laparoscopic	206 (43.3%)	29 (40.3%)	
Diverting ileostomy [*n* (%)]	204(42.9%)	25(34.7%)	0.192
Specimen extraction approaches [*n* (%)]			0.526
Conventional extraction	140 (86.1%)	60 (83.3%)	
NOSES	66 (13.9%)	12 (16.7%)	
Operation time [median (IQR), min]	155 (126.3–182.0)	170 (140–201.5)	0.026
Estimated blood loss [median (IQR), ml]	50 (40–100)	50 (50–100)	0.085
Conversion [*n* (%)]	8 (1.7%)	4 (5.6%)	0.06
Tumor differentiation [*n* (%)]			0.577
Poor	54 (11.3%)	11 (51.3%)	
Moderate	368 (77.3%)	52 (72.2%)	
High	54 (11.4%)	9 (12.5%)	
Tumor diameter [median (IQR), cm]	4 (3–5)	4 (3.5–5.5)	0.118
Harvested lymph nodes [mean (SD)]	16 (12–23)	16 (12–24)	0.637
Pathological *T* stage [*n* (%)]			0.515
T1	49 (10.3%)	9 (12.5%)	
T2	93 (19.5%)	13 (18.1%)	
T3	269 (56.5%)	36 (50.0%)	
T4	65 (13.7%)	14 (19.4%)	
Pathological *N* stage [*n* (%)]			0.691
N0	304 (63.9%)	44 (61.1%)	
N1	92 (19.3%)	17 (23.6%)	
N2	80 (16.8%)	11 (15.3%)	
Tumor stage [*n* (%)]			0.900
I	95 (20.0%)	14 (19.4%)	
II	209 (43.9%)	30 (41.7%)	
III	172 (36.1%)	28 (38.9%)	

*NOSES* natural orifice specimen extraction surgery

The postoperative characteristics of the two groups are shown in Table 3. Regarding the utilization of postoperative opioid analgesics, 44.3% of patients in the PPOI group required opioid analgesics, in contrast to 24.4% in the non-PPOI group. According to the Clavien-Dindo classification, there were no significant differences between the two groups in terms of minor complications (grades I–II). However, it is noteworthy that major complications (grades III–IV) were more commonly observed in the PPOI group. In the PPOI group, the postoperative hospital stay was significantly longer (*p* < 0.001), and inpatient costs were higher (*p* < 0.001).

**Table 3:** Postoperative characteristics of patients in the PPOI group and non-PPOI group

	Non-PPOI group (*n* = 476)	PPOI group (*n* = 72)	*p*
Postoperative use of opioid analgesic [*n* (%)]	116 (24.4%)	29 (40.3%)	0.004
Postoperative transfusion [*n* (%)]	21 (4.4%)	7 (9.7%)	0.078^a^
Time to flatus [median (IQR), days]	2 (2–2)	4 (3–5)	< 0.001
Time to stool [median (IQR), days]	3 (2–3)	5.5 (5–6)	< 0.001
Time to first tolerance of solids [median (IQR), days]	3 (2–3)	5 (4–5)	< 0.001
Abdominal distension [*n* (%)]	11 (2.3%)	71 (98.6%)	< 0.001
Nausea or vomiting [*n* (%)]	1 (0.2%)	31 (43.1%)	< 0.001
Postoperative complications [*n* (%)]			
Minor (CDC I–II)	107 (22.5%)	18 (25%)	0.635
Major (CDC III–IV)	17 (3.6%)	7 (9.7%)	0.027^a^
Postoperative hospital stay [median (IQR), days]	7 (6–8)	11 (8–15)	< 0.001
Inpatient cost [median (IQR), $]	74,997 (65,263–89,440)	84,028 (74,439.8–101,932.8)	< 0.001
30-day readmission [*n* (%)]	20 (4.2%)	3 (4.2%)	1^a^
30-day reoperation [*n* (%)]	16 (3.4%)	6 (8.3%)	0.056^a^

*CDC* Clavien-Dindo classification

^a^Using Fisher’s exact test

### Factors associated with PPOI

Univariate analysis showed that age ≥ 65 years, previous abdominal surgery, hypoproteinemia, anemia, hypokalemia, high surgical difficulty, operative time ≥ 180 min, estimated blood loss ≥ 100 ml, conversion, postoperative use of opioid analgesic, and perioperative transfusion were potential predictors of PPOI. Subsequent multivariate logistic regression analysis showed that age ≥ 65 years (OR = 1.816, 95%CI 1.040–3.172, *p* = 0.036), hypoproteinemia (OR = 2.565, 95%CI 1.183–5.563, *P* = 0.017), high surgical difficulty (OR = 2.934, 95%CI 1.406–6.121, *p* = 0.004), and postoperative use of opioid analgesic (OR = 2.624, 95%CI 1.513–4.553, *p* = 0.001) were independent predictors of PPOI (Table 4).

**Table 4:** Univariable analysis and multivariable logistic regression of PPOI

Variables	Univariate analysis		Multivariate analysis	
	OR (95% CI)	*P* value	OR (95% CI)	*P* value
Baseline characteristics				
Sex				
Female	Ref			
Male	1.331 (0.808–2.192)	0.261		
Age (years)				
< 65	Ref			
≥ 65	2.060 (1.240–3.421)	0.005	1.816 (1.040–3.172)	0.036
Smoking history				
Yes	1.128 (0.648–1.967)	0.670		
No	Ref			
Drinking history				
Yes	1.275 (0.634–2.560)	0.495		
No	Ref			
Hypertension				
Yes	1.062 (0.615–1.834)	0.829		
No	Ref			
Diabetes				
Yes	0.527 (0.204–1.362)	0.186		
No	Ref			
Respiratory disease				
Yes	0.811 (0.334–1.969)	0.643		
No	Ref			
Cardiac disease				
Yes	0.907 (0.309–2.659)	0.858		
No	Ref			
Previous abdominal surgery				
Yes	1.775 (0.997–3.158)	0.051	1.576 (0.836–6.309)	0.16
No	Ref			
ASA score				
I	Ref			
II	1.044 (0.619–1.759)	0.873		
III	0.778 (0.262–2.306)	0.650		
Hypoproteinemia				
Yes	3.465 (1.742–6.893)	< 0.001	2.565 (1.183–5.563)	0.017
No	Ref			
Anemia				
Yes	1.748 (0.983–3.110)	0.057	1.322 (0.642–2.722)	0.449
No	Ref			
Hypokalaemia				
Yes	2.333 (0.954–5.705)	0.063	2.361 (0.883–6.309)	0.087
No	Ref			
Narrow pelvis				
Yes	1.624 (0.777–3.397)	0.198		
No	Ref			
Large MFA				
Yes	1.225 (0.722–2.077)	0.452		
No	Ref			
Body mass index (kg/m^2^)				
< 25	Ref			
≥ 25	1.479 (0.900–2.431)	0.123		
Visceral obesity				
Yes	1.412 (0.822–2.426)	0.212		
No	Ref			
Sarcopenia				
Yes	1.491 (0.870–2.553)	0.146		
No	Ref			
Preoperative chemotherapy				
Yes	1.227 (0.553–2.725)	0.615		
No	Ref			
Preoperative radiotherapy				
Yes	1.400 (0.596–3.289)	0.440		
No	Ref			
Surgical difficulty				
High	3.492 (1.821–6.698)	< 0.001	2.934 (1.406–6.121)	0.004
Low	Ref			
Intraoperative characteristics				
Surgery type				
Robotic-assisted	0.884 (0.534–1.464)	0.632		
Laparoscopic	Ref			
Diverting ileostomy				
Yes	0.709 (0.422–1.191)	0.194		
No	Ref			
Specimen extraction approaches				
Conventional extraction	Ref			
NOSES	1.242 (0.634–2.433)	0.527		
Operation time (min)				
< 180	Ref			
≥ 180	1.773 (1.071–2.934)	0.026	1.427 (0.794–2.565)	0.234
Estimated blood loss (ml)				
< 100	Ref			
≥ 100	2.444 (1.206–4.956)	0.013	1.733 (0.779–3.855)	0.178
Conversion				
Yes	3.441 (1.009–11.736)	0.048	3.853 (0.995–14.925)	0.051
No	Ref			
Pathological characteristics				
Tumor differentiation				
Poor	Ref			
Moderate	0.694 (0.341–1.412)	0.313		
High	0.818 (0.314–2.133)	0.681		
Tumor height				
Low	1.372 (0.828–2.273)	0.220		
Middle	Ref			
Tumor diameter (cm)				
≤ 5	Ref			
> 5	1.649 (0.929–2.928)	0.088	0.864 (0.438–1.708)	0.675
Tumor stage				
I	Ref			
II	0.974 (0.494–1.921)	0.939		
III	1.105 (0.555–2.200)	0.777		
Postoperative characteristics				
Postoperative use of opioid analgesic				
Yes	2.849 (1.710–4.747)	< 0.001	2.624 (1.513–4.553)	0.001
No	Ref			
Postoperative transfusion				
Yes	2.333 (0.954–5.705)	0.063	1.406 (0.464–4.259)	0.547
No	Ref			

*ASA* American Society of Anesthesiologists Classification, *MFA* rectal mesenteric fat, *NOSES* natural orifice specimen extraction surgery

### Construction of a nomogram for PPOI

**Fig. 1:**
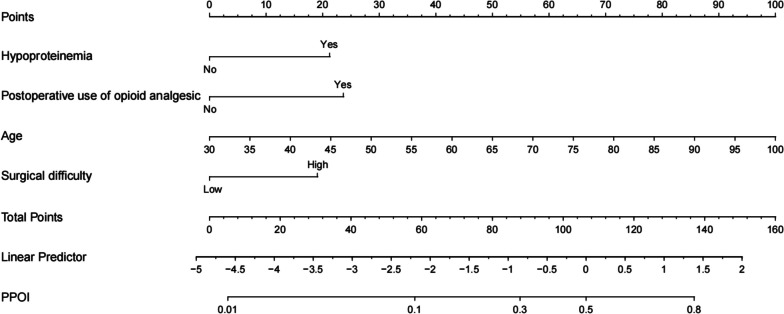
Nomogram prediction of prolonged postoperative ileus

Based on the results of multivariable logistic regression analysis, a nomogram was generated to predict the incidence of PPOI (Fig. 1). A higher total score indicated a higher likelihood of PPOI, which was calculated by summing the scores for each variable.

**Fig. 2:**
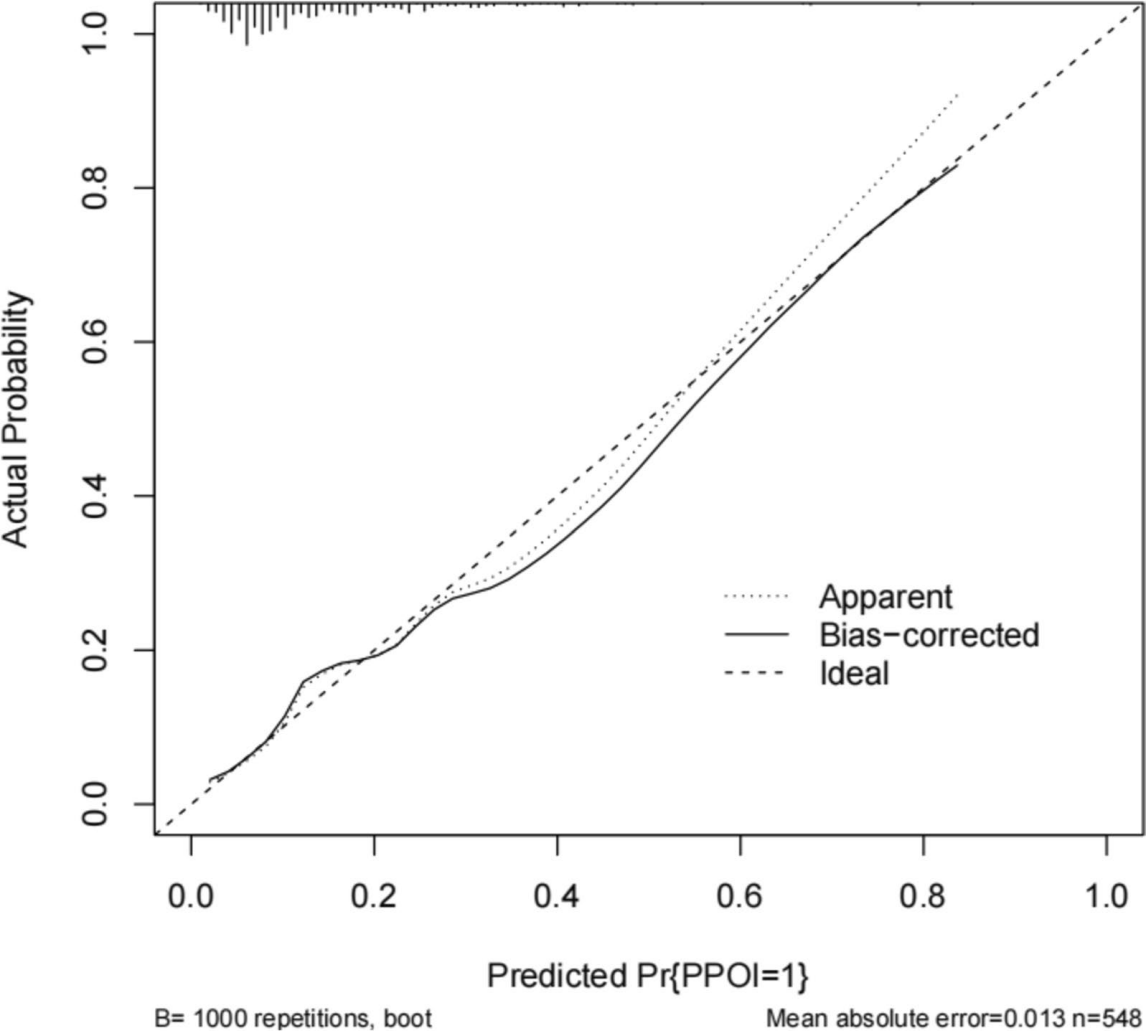
Calibration curve of the nomogram model that predicts the risk of prolonged postoperative ileus. The dashed line represents an ideal evaluation, whereas the red line represents the performance of the nomogram

**Fig. 3:**
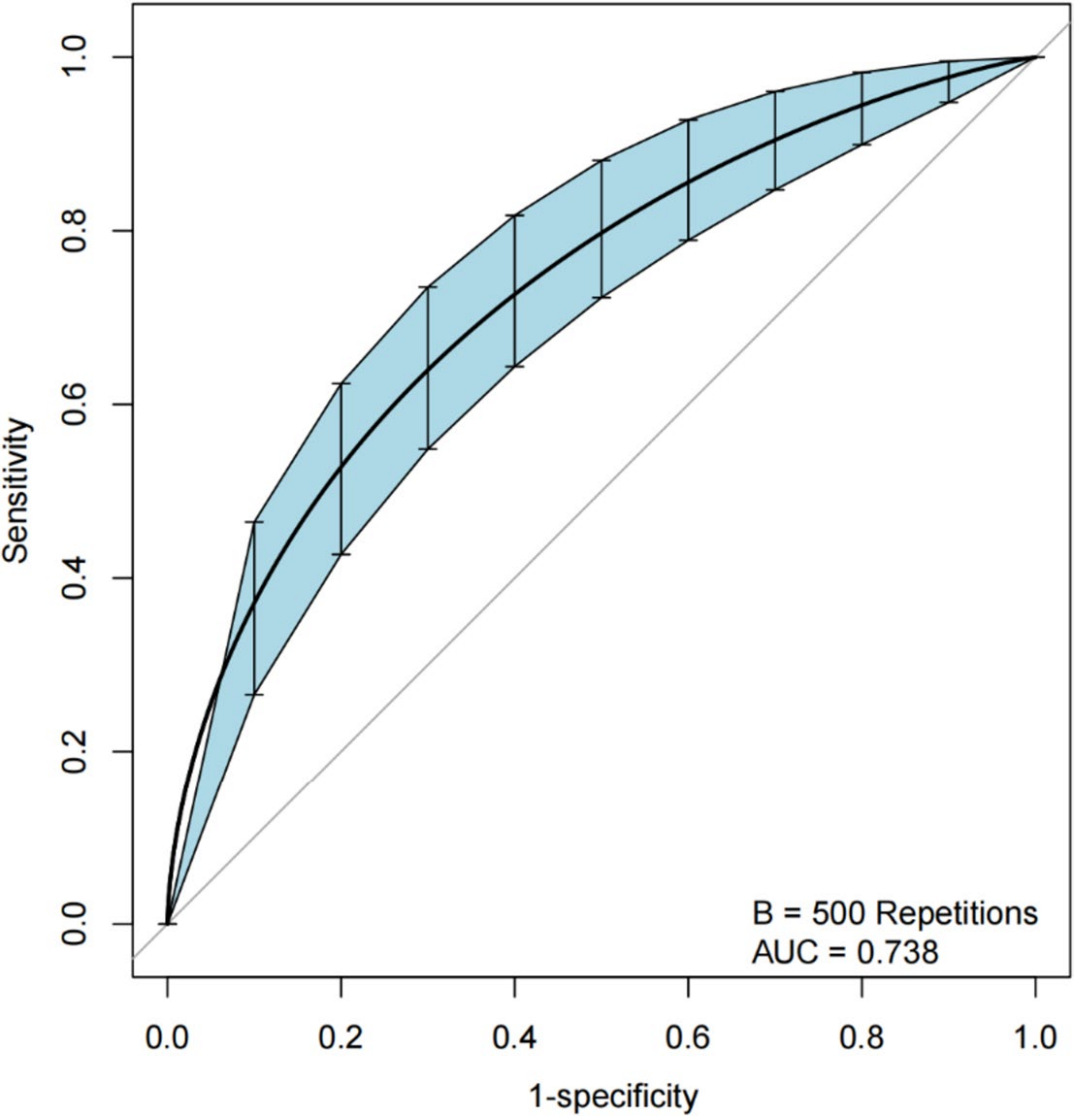
The ROC curve was measured by bootstrapping for 500 repetitions, AUC = 0.738

**Fig. 4:**
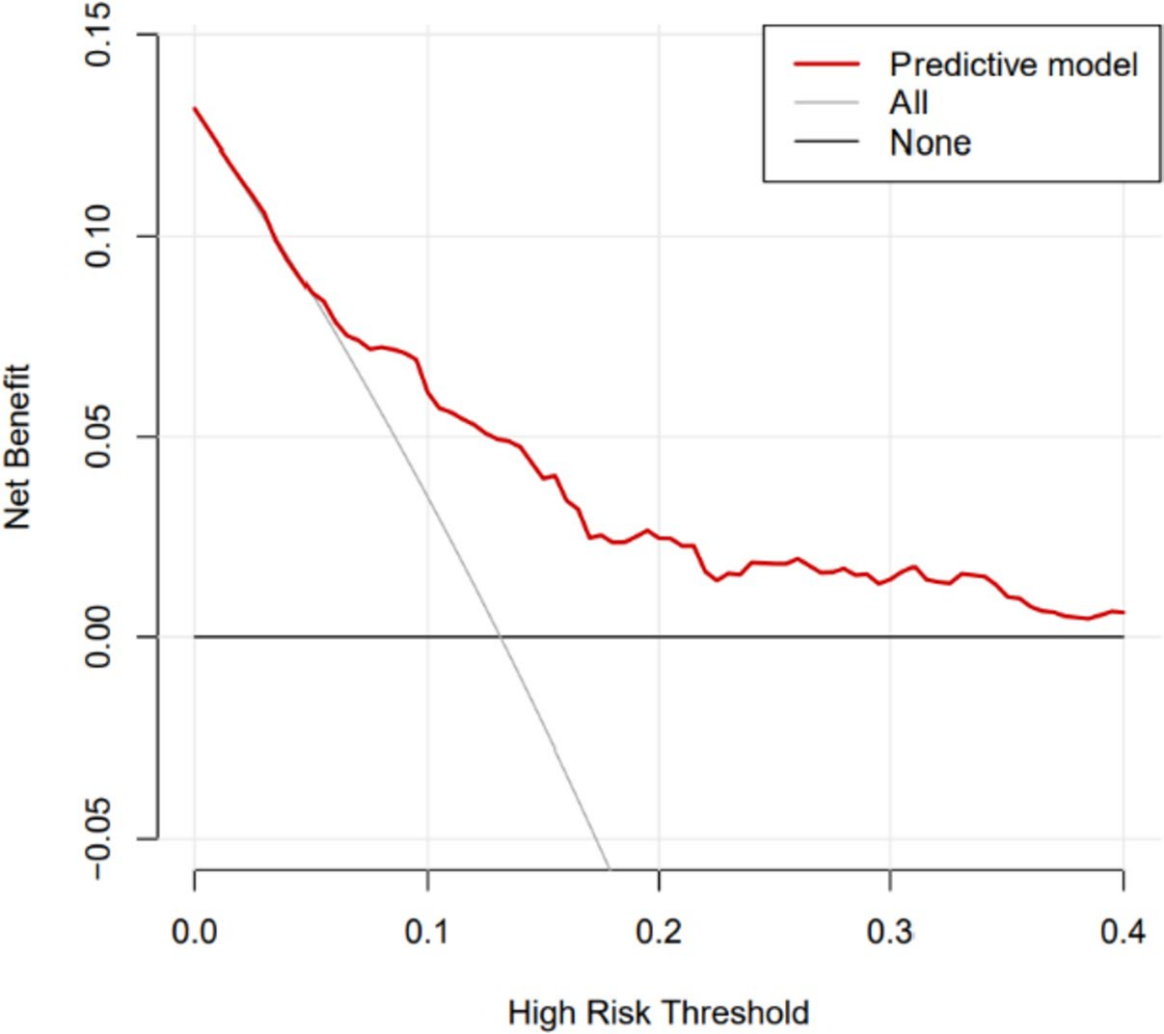
Decision curve analysis for the prediction model. The *y*-axis represents the net benefit. The *x*-axis represents the threshold probability. The gray and black lines represent the assumption that all and none of the patients had long-term disease-free survival

The bias-corrected calibration plot with 500-sample bootstrapping for the prediction model demonstrated satisfactory consistency (Fig. 2). Similarly, the discriminative ability of the model was evaluated using the bias-corrected AUC, which was estimated using bootstrap resampling with 500 iterations. The calculated AUC was found to be 0.738 (95%CI 0.736–0.741) (Fig. 3). Furthermore, the decision curve demonstrates that utilizing the nomogram to predict the probability of PPOI provides more benefit than either the treat-all-patients scheme or the treat-none scheme, indicating that the nomogram has clinical value (Fig. 4).

## Discussion

Currently, the concept of enhanced recovery after surgery is widely accepted by surgeons. PPOI as a common complication that hinders postoperative recovery has received widespread attention. In the present study, 13.1% of the patients were diagnosed with PPOI, which is comparable to the 15% occurrence of PPOI following laparoscopic rectal surgery reported by Elisabeth et al. [7]. Liang et al. reported an incidence of PPOI of 19.75% in gastrectomy for gastric cancer [5], while Lind et al. showed an incidence of 10.2% by analyzing 1254 patients with colorectal cancer [10].

In this study, we evaluated the relationship between clinical characteristics and the occurrence of PPOI in patients who underwent laparoscopic anterior resection for rectal cancer. Age ≥ 65 years, hypoproteinemia, high surgical difficulty, and postoperative use of opioid analgesic have been proven to be significantly correlated with PPOI. The selected predictors were then used to construct a nomogram that could help identify patients at risk of PPOI. In addition, it has been confirmed that this nomogram has a good diagnostic performance and has been validated internally. Understanding the clinical factors that predispose to PPOI is the first step in developing tools that can help predict its occurrence. This, in turn, may help to identify individuals at risk and allow early intervention to mitigate or terminate episodes.

In this study, a significant correlation was observed between hypoproteinemia and PPOI, this finding is similar to a study by Liang et al. [9]. Hypoalbuminemia is common in patients with gastrointestinal cancers, primarily attributed to dietary deficiencies, impaired liver function, increased loss of ascites, and gastrointestinal bleeding. Hypoproteinaemia may lead to intestinal edema, which affects the recovery of intestinal function [16]. Furthermore, hypoalbuminemia has also been reported to be an indicator of deterioration in performance status or tumor progression [17]. Several studies have also demonstrated that preoperative albumin levels can be predictive of surgical risk and postoperative complications [18–20].

Of note, the multivariable analysis also indicated that advanced age was an independent risk factor for PPOI, which is consistent with several previous studies [5, 7]. This observation could be attributed to the fact that older adults tend to have a higher prevalence of medical comorbidities, clinical frailty, and relatively poorer nutritional and functional statuses compared to younger adults [21]. Our study emphasizes the necessity of perioperative dietary interventions for older patients and those with hypoalbuminemia.

Vather et al. demonstrated that high surgical difficulty, as self-assessed by the surgeon, is a risk factor for developing PPOI after colorectal surgery [16]. In this study, we assessed the difficulty of surgery based on factors that have been previously reported to influence surgical difficulty [22–24]. It is worth noting that this method is more objective than a surgeon’s self-assessment. In this study, we also found high surgical difficulty is an independent risk factor for PPOI. Operation in patients with high surgical difficulty, exposure, resection, and anastomosis will be more challenging. Specifically, performing the procedure in a narrow pelvis may increase the risk of rectal wall or vascular trauma [25].

Opioids are commonly used for pain management after surgery, which is highly effective in treating both acute and chronic pain. However, opioid therapy also affects bowel function by causing opioid-induced bowel dysfunction [26]. Opioids can cause inhibition of water and electrolyte excretion and enhanced non-propulsive contractions through activation of μ-receptors located in the enteric nervous system [27]. The relationship between opioids and PPOI has been well characterized in previous studies [28, 29]. Our study also confirms that patients using opioids have a higher risk of PPOI. The peripherally acting μ-receptor antagonists such as methylnaltrexone and alvimopan are designed to block the side effects of opioids in the gastrointestinal tract while preserving the pain-relieving effects of opioids [25]. These drugs are expected to be utilized in the prevention of PPOI.

Prolonged postoperative ileus (PPOI) is a common complication after colorectal surgery, leading to an increased risk of complications, extended hospitalization, and significant financial burdens for healthcare facilities [30, 31]. Individualized treatment has been gradually emphasized in current clinical practice. In patients at higher risk of PPOI, strategies such as minimizing surgical trauma, optimizing fluid management, reducing opioid use, encouraging early physical activity and promoting gum chewing have been reported as effective measures to prevent PPOI [32]. Additionally, in these patients, special care should be taken in postoperative monitoring to prevent aspiration pneumonia and PPOI-related death [33].

This study has several limitations. First, this study is retrospective in nature, and the sample size was relatively small. Second, this model lacks external validation, and to address this limitation, we have employed bootstrap resampling for internal validation. Despite the abovementioned limitations, this study boasts several notable advantages. To the best of our knowledge, this is the first nomogram specifically designed to predict PPOI after laparoscopic low anterior resection for rectal cancer. Furthermore, we conducted measurements of patients’ pelvic and body composition, facilitating a more comprehensive assessment of surgical difficulty and the nutritional status of the patients.

## Conclusion

We created a novel nomogram for predicting PPOI after laparoscopic low anterior resection. This nomogram can assist surgeons in identifying patients at a heightened risk of PPOI.

## Data Availability

The data that support the findings of this study are available from the corresponding author on reasonable request.

## Abbreviations

PPOI: Prolonged postoperative ileus
CT: Computed tomography
VAT: Visceral adipose tissue area
SMA: Skeletal muscle area
MFA: Mesorectal fat area
DCA: Decision curve analysis
ROC: Receiver operating characteristic
ASA: American Society of Anesthesiologists Classification
NOSES: Natural orifice specimen extraction surgery
CDC: Clavien-Dindo Classification
SD: Standard deviation
IQR: Interquartile range
